# Auscultation and Percussion

**Published:** 1891-04

**Authors:** 


AUSCULTATION AND PERCUSSION. By Frederick C. Shattuck, M. D., is another interesting volume added to that popular series—The Physician's Leisure Library—and well fills the scope it is intended to, that is to familiarize the reader with—first, the healthy condition of the chest, that he may better appreciate the diseased conditions when described or met with in practice.

The book is concise, and well worth the perusal of the physician during his leisure moments.

